# Supplementary material for: Anthropozoonotic spillovers reveal sustained long-term cryptic circulation of SARS-CoV-2 within and between Lithuanian mink farms
Source: Virus Evol. 2026 Mar 13;12(1):veag014. doi: 10.1093/ve/veag014 (PMC13082393; doi:10.1093/ve/veag014)
Supplement: Mink_paper_supplementary_VE_No_figures_HumSoc_veag014 [file mink_paper_supplementary_ve_no_figures_humsoc_veag014.docx]

# Supplementary materials

## SARS-CoV-2 genomic surveillance programme

Briefly, the genomic surveillance programme of Lithuania combined the sequencing capacity of Vilnius University Life Sciences Center, Vilnius University Hospital Santaros Klinikos, Lithuanian University of Health Sciences, Lithuanian University of Health Sciences Hospital Kauno Klinikos, and the European Center for Disease Control (ECDC). For details about each institution’s sequencing infrastructure and bioinformatic processing see [(Dudas et al. 2021)](https://www.zotero.org/google-docs/?krWDXk). This SARS-CoV-2 genomic surveillance programme averaged over 600 genomes per week (translating to the ability to exclude the circulation of a lineage at a weekly 0.005 frequency with 0.95 probability [(Brito et al. 2022)](https://www.zotero.org/google-docs/?oDl7ie)). From the inception of this programme in March 2021, by sampling design, all SARS-CoV-2 PCR-positive samples are directed to sequencing if a person is registered as working on a fur farm. The SFVS had no specific genomic surveillance design for mink and sequencing was only done in response to confirmed outbreaks in mink farms.

### Mink-origin SARS-CoV-2 genomes

SARS-CoV-2 samples from mink were collected by the SFVS and sequenced by the National Food and Veterinary Service of Lithuania or Erasmus University Medical Center. These were mapped to a reference SARS-CoV-2 genome using Dragen (Version 3.5.0 - 3.5.4, Illumina) and submitted to GISAID [(Khare et al. 2021)](https://www.zotero.org/google-docs/?5dC381). Upon inspection, the quality of these genomes was found to be insufficient, and they were remapped from raw FASTQ files using COVID-19-SIGNAL pipeline [(Nasir et al. 2024)](https://www.zotero.org/google-docs/?SxObZS) under default parameters. A total of 58 mink samples were used for this study, with Pangolin [(O’Toole et al. 2021)](https://www.zotero.org/google-docs/?RVams1) assigning the 58 genomes to 6 broader lineages (see next section): 30 to AY.4, 4 to AY.122, 3 to B.1.1.7, 9 to B.1.1.280, 5 to B.1.177.60, and 7 to B.1.343 (Supplementary material file “Sample_table_with_EPI_ISL.csv”).

## Contextual data

Contextual data for molecular clock analyses were generated on a lineage-by-lineage basis (split into B.1.1.7, AY.4, AY.122, B.1.177.60, B.1.1.280, and B.1.343 categories) by uploading the reassembled mink-origin SARS-CoV-2 genomes of each lineage to UShER [(Turakhia et al. 2021)](https://www.zotero.org/google-docs/?ZKDpom) and noting the genomes most closely related to these focal sequences which we called the “mink-adjacent” sequences. For broader context, a metadata file was downloaded from GISAID for all sequences on the database [(Shu and McCauley 2017)](https://www.zotero.org/google-docs/?qFXEHA). For each sequence in the database, we assigned one of our six focal lineage categories, i.e. if a sequence was assigned to lineage Q.1 or Q.2 (i.e., B.1.1.7.1 or B.1.1.7.2) it was considered to be in the B.1.1.7 category. Due to occasional lineage misassignments on GISAID, every sequence belonging to a given lineage had to fall within a known circulation window of that lineage (B.1.1.7: 2020 Oct–2021 Oct, AY.4: 2021 May–2022 Feb, AY.122: 2021 May–2022 Feb, B.1.177.60: 2020 Oct–2021 May, B.1.1.280: 2020 Aug–2021 Mar, B.1.343: 2020 Mar–2020 Sep). Due to different lineage prevalences (globally and locally), we chose different numbers to sample at random from each lineage category (1 sequence per country per month for B.1.1.7, AY.4 and AY.122, 5 sequences per country per month for B.1.177.60, and 10 sequences per country per month for B.1.1.280 and B.1.343). For some lineages, 1 sequence per country per month resulted in datasets that were too large to analyse in BEAST, and in those cases, we randomly downsampled, by randomly removing contextual genomes from the dataset, reducing them to 40% (B.1.1.7), 50% (AY.122), or 60% (AY.4) of their original size. Due to low numbers of B.1.1.280 and B.1.343 lineage genomes overall, additional B.1.1 and B.1 sequences (respective ancestral lineages), respectively, were added to each category from GISAID, with 25 B.1.1 sequences per month added to the B.1.1.280 category and 35 B.1 sequences per month added to the B.1.343 category. After contextual sequence generation, the sequences were aligned using Nextclade version 3.8.2 [(Aksamentov et al. 2021)](https://www.zotero.org/google-docs/?HZZgzZ) to reference genome NC_045512.2.

## Phylogenetic analysis

Each of the six lineage datasets (B.1.1.7 (286 genomes), AY.4 (276 genomes), AY.122 (258 genomes), B.1.177.60 (249 genomes), B.1.1.280 (144 genomes), and B.1.343 (190 genomes)) was used for analysis with BEAST v1.10.4. XML files for these analyses were generated using BEAUti v1.10.4. with the following parameters: 1) SRD06 substitution model [(Shapiro, Rambaut, and Drummond 2006)](https://www.zotero.org/google-docs/?dpxMte) that models substitutions separately depending on codon position (one model for the first two codon positions and another for the third position) according to two independent Hasegawa-Kishino-Yano (HKY) + Γ_4_ substitution models [(Hasegawa, Kishino, and Yano 1985; Yang 1994)](https://www.zotero.org/google-docs/?6qtZT9) with gene coordinates provided in the XML. The HKY model assumes different substitution rates for transitions and transversions and unequal nucleotide frequencies, while Γ assumes rate heterogeneity across sites with a gamma distribution discretised into 4 rate categories; 2) an uncorrelated relaxed clock with log-normally distributed rates calibrated on tip dates [(Drummond et al. 2006)](https://www.zotero.org/google-docs/?34vXWm) and a continuous time Markov Chain reference prior [(Ferreira and Suchard 2008)](https://www.zotero.org/google-docs/?hW7DsH) on the molecular clock rate. A relaxed clock allows substitution rates to vary among branches in the tree, and in uncorrelated clock models the substitution rates are not correlated across neighboring branches. Each sequence is associated with a specific sampling date, therefore, the molecular clock is tip-calibrated; 3) Coalescent Bayesian SkyGrid tree prior [(Gill et al. 2013)](https://www.zotero.org/google-docs/?Ia0FqS) that allows for a varying effective population size across different time periods. The default gamma prior with shape 0.001 and scale of 1000 was used, favoring low population sizes with high variability. The SkyGrid number of grid points was set to 9 time points and the cut off point to 1.5 years prior to the most recent tip date; 4) 200 million steps of MCMC with sampling every 20,000 steps. For each lineage, three replicate chains (11 for B.1.1.280 lineage, explained in “Post-processing of MCMC samples” section) were run and convergence assessed using Tracer v.1.7.2 [(Rambaut et al. 2018)](https://www.zotero.org/google-docs/?YUsqEa). Convergence was assessed based on all parameters having effective sample sizes (ESSs) above 200; 5) Host information was included as a discrete trait and reconstructed using an asymmetric substitution model inferred by Bayesian stochastic search variable selection (BSSVS) [(Gill et al. 2013)](https://www.zotero.org/google-docs/?6MXifQ). To obtain Markov jumps (transitions between human and mink as a host), we used complete history logging [(Gong, Suchard, and Bloom 2013; Minin and Suchard 2008)](https://www.zotero.org/google-docs/?TMaWrQ), generating an MCMC output file with history parameters for nodes and leaves, containing Markov jump dates. 6) As we wanted to estimate the number of SARS-CoV-2 introductions into mink, we set the host trait of five human-origin samples as mink to assist ancestral state reconstruction: S21L465|Lithuania (EPI_ISL_7083492), S21L477|Lithuania (EPI_ISL_7082794), S21E887|Lithuania (EPI_ISL_2428956), S21E881|Lithuania (EPI_ISL_2428882), IBT-LCS-VU_r24_28|Lithuania (EPI_ISL_5390697). These samples are exceedingly likely to come from mink-to-human spillovers as they contain mink adaptive mutations and were not detected in the general human population at the time yet strongly and incorrectly inform ancestral state reconstruction due to lack of samples from mink. This is discussed in greater detail in results. 7) Sequences in the XML file were modified by inserting two ‘N’ nucleotide symbols after position 13 468 to bring ORF1b into frame with ORF1a.

## Post-processing of MCMC samples

For each BEAST analysis, three replicate chains were run to ensure convergence to the posterior distribution. The one exception were analyses of lineage B.1.1.280, where the posterior distribution appeared to be bimodal for parameters related to the evolutionary rate - age(root), treeLength, default.ucld.mean, default.meanRate and skygrid. We ran B.1.1.280 XMLs an additional eight times with the same bimodal behaviour. Due to this bimodal behavior, we decided to use only 6 of the 11 runs (with age(root) around 2020.07 and treeLength around 47.9 years), with a burn-in of 75% (150 million states) to generate a workable dataset. All log and tree files are provided in supplementary materials.

## Biosecurity requirements in Lithuanian mink farms

Biosecurity requirements issued by the SFVS have been compulsory on mink farms in the country since 2015 [(Fenollar et al. 2021)](https://www.zotero.org/google-docs/?0sMwD5). The main requirements were: 1) fencing of farms to keep out wildlife and unauthorized persons (likely ineffective [(Rasmussen et al. 2024)](https://www.zotero.org/google-docs/?HiyOa0)), 2) staff must wear only mink dedicated work clothes and footwear, 3) use of disinfection barriers, handwashing and hand disinfection equipment, 4) regular rodent and pest control, in addition to disinfection of vehicles while entering and exiting the premises through changing rooms with showers were recommended. Furthermore, the responsible person on a mink farm has to be informed if a visitor has visited other mink premises in the last 48 hours, has been on a fox or raccoon dog hunt, has been in contact with animal by-products, in which case the person responsible for the mink farm will decide whether such a visitor may be admitted to the holding. However, it is unclear how compliance with these requirements is regulated.

## Description of statistical data

### Kernel Density Estimation (KDE)

We used Python script to calculate KDE from posterior distributions of BEAST data regarding human-to-mink transition dates for each lineage. We employed Gaussian KDE from the scipy package (scipy.stats.gaussian_kde) with bandwidth modified by 0.8 (kde.set_bandwidth(bw_method=kde.factor * 0.8). We set the evaluation at a thousand points for each case (x_kde = np.linspace(min(data), max(data), 1000)) and calculating each value on Y-axis (y_kde = kde(x_kde)) with further normalization for more even visualization, based on total area under the curve (dx = x_kde[1] - x_kde[0]; y_kde_normalized = y_kde / (np.sum(y_kde) * dx * 2000))

### Highest Density Region (HDR) estimation

We used HDR calculation regarding transition from human to mink event number and dates for each lineage. We used a custom Python script, by first using Gaussian KDE (scipy.stats.gaussian_kde) to count and bin x-values spanning the sample range, by creating a probability density function. HDR was defined as a union of shortest intervals that together contain 95% of the probability density.

## Additional alignments

## **Root-to-tip regressions**

Maximum-likelihood phylogenies were inferred from SARS-CoV-2 genome alignments using IQ-TREE [(Nguyen et al. 2015)](https://www.zotero.org/google-docs/?P7BJoS) (version 3.0.1) package, with the HKY substitution model and gamma-distributed rate variation discretised into four categories (HKY+Γ4) [(Hasegawa, Kishino, and Yano 1985; Yang 1994)](https://www.zotero.org/google-docs/?avtyJz). The trees were rooted using a root-to-tip regression method, in which the root position is optimized to maximize the coefficient of determination (R²) between tip sampling dates and genetic divergence from the root by using the baltic package (Dev-main branch, checked out 2026 Jan 28).

# References

[Aksamentov, Ivan, et al. (2021) ‘Nextclade: Clade Assignment, Mutation Calling and Quality Control for Viral Genomes’, *Journal of Open Source Software*, 6/67: 3773.](https://www.zotero.org/google-docs/?5CCft2)

[Brito, Anderson F., et al. (2022) ‘Global Disparities in SARS-CoV-2 Genomic Surveillance’, *Nature Communications*, 13/1: 7003.](https://www.zotero.org/google-docs/?5CCft2)

[Drummond, Alexei J., et al. (2006) ‘Relaxed Phylogenetics and Dating with Confidence’, *PLoS Biology*, 4/5: e88.](https://www.zotero.org/google-docs/?5CCft2)

[Dudas, Gytis, et al. (2021) ‘Emergence and Spread of SARS-CoV-2 Lineage B. 1.620 with Variant of Concern-like Mutations and Deletions’, *Nature Communications*, 12/1: 5769.](https://www.zotero.org/google-docs/?5CCft2)

[Fenollar, Florence, et al. (2021) ‘Mink, SARS-CoV-2, and the Human-Animal Interface’, *Frontiers in Microbiology*, 12: 663815.](https://www.zotero.org/google-docs/?5CCft2)

[Ferreira, Marco AR, and Marc A. Suchard (2008) ‘Bayesian Analysis of Elapsed Times in Continuous‐time Markov Chains’, *Canadian Journal of Statistics*, 36/3: 355–68.](https://www.zotero.org/google-docs/?5CCft2)

[Gill, Mandev S., et al. (2013) ‘Improving Bayesian Population Dynamics Inference: A Coalescent-Based Model for Multiple Loci’, *Molecular Biology and Evolution*, 30/3: 713–24.](https://www.zotero.org/google-docs/?5CCft2)

[Gong, Lizhi Ian, Marc A. Suchard, and Jesse D. Bloom (2013) ‘Stability-Mediated Epistasis Constrains the Evolution of an Influenza Protein’, *Elife*, 2: e00631.](https://www.zotero.org/google-docs/?5CCft2)

[Hasegawa, Masami, Hirohisa Kishino, and Taka-aki Yano (1985) ‘Dating of the Human-Ape Splitting by a Molecular Clock of Mitochondrial DNA’, *Journal of Molecular Evolution*, 22: 160–74.](https://www.zotero.org/google-docs/?5CCft2)

[Khare, Shruti, et al. (2021) ‘GISAID’s Role in Pandemic Response’, *China CDC Weekly*, 3/49: 1049.](https://www.zotero.org/google-docs/?5CCft2)

[Minin, Vladimir N., and Marc A. Suchard (2008) ‘Fast, Accurate and Simulation-Free Stochastic Mapping’, *Philosophical Transactions of the Royal Society B: Biological Sciences*, 363/1512: 3985–95.](https://www.zotero.org/google-docs/?5CCft2)

[Nasir, Jalees A., et al. (2024) ‘SARS-CoV-2 Illumina GeNome Assembly Line (SIGNAL), a Snakemate Workflow for Rapid and Bulk Analysis of Illumina Sequencing of SARS-CoV-2 Genomes’, *NAR Genomics and Bioinformatics*, 6/4: lqae176.](https://www.zotero.org/google-docs/?5CCft2)

[Nguyen, Lam-Tung, et al. (2015) ‘IQ-TREE: A Fast and Effective Stochastic Algorithm for Estimating Maximum-Likelihood Phylogenies’, *Molecular Biology and Evolution*, 32/1: 268–74, https://doi.org/10.1093/molbev/msu300.](https://www.zotero.org/google-docs/?5CCft2)

[O’Toole, Áine, et al. (2021) ‘Assignment of Epidemiological Lineages in an Emerging Pandemic Using the Pangolin Tool’, *Virus Evolution*, 7/2: veab064.](https://www.zotero.org/google-docs/?5CCft2)

[Rambaut, Andrew, et al. (2018) ‘Posterior Summarization in Bayesian Phylogenetics Using Tracer 1.7’, *Systematic Biology*, 67/5: 901–4.](https://www.zotero.org/google-docs/?5CCft2)

[Rasmussen, Thomas Bruun, et al. (2024) ‘Emergence and Spread of SARS-CoV-2 Variants from Farmed Mink to Humans and Back during the Epidemic in Denmark, June-November 2020’, *Plos Pathogens*, 20/7: e1012039.](https://www.zotero.org/google-docs/?5CCft2)

[Shapiro, Beth, Andrew Rambaut, and Alexei J. Drummond (2006) ‘Choosing Appropriate Substitution Models for the Phylogenetic Analysis of Protein-Coding Sequences’, *Molecular Biology and Evolution*, 23/1: 7–9.](https://www.zotero.org/google-docs/?5CCft2)

[Shu, Yuelong, and John McCauley (2017) ‘GISAID: Global Initiative on Sharing All Influenza Data–from Vision to Reality’, *Eurosurveillance*, 22/13: 30494.](https://www.zotero.org/google-docs/?5CCft2)

[Turakhia, Yatish, et al. (2021) ‘Ultrafast Sample Placement on Existing tRees (UShER) Enables Real-Time Phylogenetics for the SARS-CoV-2 Pandemic’, *Nature Genetics*, 53/6: 809–16.](https://www.zotero.org/google-docs/?5CCft2)

[Yang, Ziheng (1994) ‘Maximum Likelihood Phylogenetic Estimation from DNA Sequences with Variable Rates over Sites: Approximate Methods’, *Journal of Molecular Evolution*, 39: 306–14.](https://www.zotero.org/google-docs/?5CCft2)

## **Figure and table legends**

**Table S1.** Exact 95% HPD ranges for human-to-mink transition events.

**Table S2.** Summary of SARS-CoV-2 Lineage Dataset Used for Phylogenetic Analyses.

**Figure S1. SNP alignments with phylogenetic trees.** Starting from the left: A condensed alignment of polymorphic sites is displayed, keeping only the mutations in mink samples for lineage AY.4 (One of multiple alignments). Further to the right, subtrees containing the mink samples, extracted from MCC trees (with the closest contextual samples). A map of Lithuania with lines from the leaf in a tree to a point on a map, representing the municipality of origin of a given sample (points are randomly positioned within the area of the municipality). Tips are coloured according to sample type: mink farm worker (yellow), general Lithuanian population (green), mink (red), or sequences outside of Lithuania (gray), see legend (upper right corner). The coats of arms of municipalities where mink samples were collected are displayed below the tree with municipalities coloured same as the background of each municipal coat of arms.

**Figure S2. SNP alignments with phylogenetic trees.** Starting from the left: A condensed alignment of polymorphic sites is displayed, keeping only the mutations in mink samples for lineage AY.4 (One of multiple alignments). Further to the right, subtrees containing the mink samples, extracted from MCC trees (with the closest contextual samples). Additionally, a map of Lithuania with lines from the leaf in a tree to a point on a map, representing the municipality of origin of a given sample (points are randomly positioned within the area of the municipality if municipality data is available). Tips are coloured according to sample type: mink farm worker (yellow), general Lithuanian population (green), mink (red), or sequences outside of Lithuania (gray), see legend (upper right corner). The coats of arms of municipalities where mink samples were collected are displayed below the tree with municipalities coloured same as the background of each municipal coat of arms.

**Figure S3. SNP alignments with phylogenetic trees.** Starting from the left: A condensed alignment of polymorphic sites is displayed, keeping only the mutations in mink samples for lineage AY.4 (One of multiple alignments). Further to the right, subtrees containing the mink samples, extracted from MCC trees (with the closest contextual samples). Additionally, a map of Lithuania with lines from the leaf in a tree to a point on a map, representing the municipality of origin of a given sample (points are randomly positioned within the area of the municipality). Tips are coloured according to sample type: mink farm worker (yellow), general Lithuanian population (green), mink (red), or sequences outside of Lithuania (gray), see legend (upper right corner). The coats of arms of municipalities where mink samples were collected are displayed below the tree with municipalities coloured same as the background of each municipal coat of arms.

**Figure S4. SNP alignments with phylogenetic trees.** Starting from the left: A condensed alignment of polymorphic sites is displayed, keeping only the mutations in mink samples for lineage AY.4 (One of multiple alignments). Further to the right, subtrees containing the mink samples, extracted from MCC trees (with the closest contextual samples). Additionally, a map of Lithuania with lines from the leaf in a tree to a point on a map, representing the municipality of origin of a given sample (points are randomly positioned within the area of the municipality). Tips are coloured according to sample type: mink farm worker (yellow), general Lithuanian population (green), mink (red), or sequences outside of Lithuania (gray), see legend (upper right corner). The coats of arms of municipalities where mink samples were collected are displayed below the tree with municipalities coloured same as the background of each municipal coat of arms.

**Figure S5. SNP alignments with phylogenetic trees.** Starting from the left: A condensed alignment of polymorphic sites is displayed, keeping only the mutations in mink samples for lineage AY.122. Further to the right, subtrees containing the mink samples, extracted from MCC trees (with the closest contextual samples). Additionally, a map of Lithuania with lines from the leaf in a tree to a point on a map, representing the municipality of origin of a given sample (points are randomly positioned within the area of the municipality). Tips are coloured according to sample type: mink farm worker (yellow), general Lithuanian population (green), mink (red), or sequences outside of Lithuania (gray), see legend (upper right corner). The coats of arms of municipalities where mink samples were collected are displayed below the tree with municipalities coloured same as the background of each municipal coat of arms.

**Figure S6. SNP alignments with phylogenetic trees.** Starting from the left: A condensed alignment of polymorphic sites is displayed, keeping only the mutations in mink samples for lineage B.1.1.7. (One of two alignments). Further to the right, subtrees containing the mink samples, extracted from MCC trees (with the closest contextual samples). Additionally, a map of Lithuania with lines from the leaf in a tree to a point on a map, representing the municipality of origin of a given sample (points are randomly positioned within the area of the municipality if municipality data is available). Tips are coloured according to sample type: mink farm worker (yellow), general Lithuanian population (green), mink (red), or sequences outside of Lithuania (gray), see legend (upper right corner). The coats of arms of municipalities where mink samples were collected are displayed below the tree with municipalities coloured same as the background of each municipal coat of arms.

**Figure S7. SNP alignments with phylogenetic trees.** Starting from the left: A condensed alignment of polymorphic sites is displayed, keeping only the mutations in mink samples for lineage B.1.1.7. (One of two alignments). Further to the right, subtrees containing the mink samples, extracted from MCC trees (with the closest contextual samples). Additionally, a map of Lithuania with lines from the leaf in a tree to a point on a map, representing the municipality of origin of a given sample (points are randomly positioned within the area of the municipality). Tips are coloured according to sample type: mink farm worker (yellow), general Lithuanian population (green), mink (red), or sequences outside of Lithuania (gray), see legend (upper right corner). The coats of arms of municipalities where mink samples were collected are displayed below the tree with municipalities coloured same as the background of each municipal coat of arms.

**Figure S8. SNP alignments with phylogenetic trees.** Starting from the left: A condensed alignment of polymorphic sites is displayed, keeping only the mutations in mink samples for lineage B.1.1.280. Further to the right, subtrees containing the mink samples, extracted from MCC trees (with the closest contextual samples). Additionally, a map of Lithuania with lines from the leaf in a tree to a point on a map, representing the municipality of origin of a given sample (points are randomly positioned within the area of the municipality). Tips are coloured according to sample type: mink farm worker (yellow), general Lithuanian population (green), mink (red), or sequences outside of Lithuania (gray), see legend (upper right corner). The coats of arms of municipalities where mink samples were collected are displayed below the tree with municipalities coloured same as the background of each municipal coat of arms.

**Figure S9. SNP alignments with phylogenetic trees.** Starting from the left: A condensed alignment of polymorphic sites is displayed, keeping only the mutations in mink samples for lineage B.1.177.60 secondary transitions. Further to the right, subtrees containing the mink samples, extracted from MCC trees (with the closest contextual samples). Additionally, a map of Lithuania with lines from the leaf in a tree to a point on a map, representing the municipality of origin of a given sample (points are randomly positioned within the area of the municipality if municipality data is available). Tips are coloured according to sample type: mink farm worker (yellow), general Lithuanian population (green), mink (red), or sequences outside of Lithuania (gray), see legend (upper right corner). The coats of arms of municipalities where mink samples were collected are displayed below the tree with municipalities coloured same as the background of each municipal coat of arms.

**Figure S10. Regression of B.1.343 ML tree.** Each point represents an individual genome, coloured by sample category (legend in lower right). The y-axis shows genetic divergence of the genome from the inferred root (substitutions per site), and the x-axis shows sampling date expressed as decimal date. The top left inset reports the slope and intercept of the regression line, as well as the Pearson correlation coefficient (*r*).

**Figure S11. Regression curve of B.1.177.60 ML tree.** Each point represents an individual genome, coloured by sample category (legend in lower right). The y-axis shows genetic divergence of the genome from the inferred root (substitutions per site), and the x-axis shows sampling date expressed as decimal date. The top left inset reports the slope and intercept of the regression line, as well as the Pearson correlation coefficient (*r*).
