# Supplementary material for: Anthropozoonotic spillovers reveal sustained long-term cryptic circulation of SARS-CoV-2 within and between Lithuanian mink farms
Source: Virus Evol. 2026 Mar 13;12(1):veag014. doi: 10.1093/ve/veag014 (PMC13082393; doi:10.1093/ve/veag014)
Supplement: vevolu_veag014 [file vevolu_veag014.docx]

Anthropozoonotic spillovers reveal sustained long-term cryptic circulation of SARS-CoV-2 within and between Lithuanian mink farms

Martynas Smičius^1^,
Ingrida Olendraitė^2,3^,
Jonas Bačelis^4^,
Aistis Šimaitis^5^,
Miglė Gabrielaitė^1,6^,
Bas B Oude Munnink^7^,
Reina S Sikkema^7^,
Arūnas Stankevičius^8^,
Žygimantas Janeliūnas^9^,
Paulius Bušauskas^10^,
Egidijus Pumputis^9^,
Simona Pilevičienė^9^,
Petras Mačiulskis^9^,
Marius Masiulis^10,11^,
Vidmantas Paulauskas^9^,
Snieguolė Ščeponavičienė^9^,
Monika Katėnaitė^2^,
Rimvydas Norvilas^2,12^,
Ligita Raugienė^2^,
Rimvydas Jonikas^13^,
Inga Nasvytienė^13^,
Živilė Žemeckienė^13^,
Kamilė Tamušauskaitė^14^,
Milda Norkienė^1^,
Emilija Vasiliūnaitė^1^,
Danguolė Žiogienė^1^,
Albertas Timinskas^1^,
Marius Šukys^13,15^,
Mantas Šarauskas^13^,
Dovilė Juozapaitė^2^,
Daniel Naumovas^2^,
Arnoldas Pautienius^16^,
Astra Vitkauskienė^17^,
Rasa Ugenskienė^13,15^,
Alma Gedvilaitė^1^,
Darius Čereškevičius^13,14^,
Laimonas Griškevičius^2^,
Marion Koopmans^7^,
Alvydas Malakauskas^18^,
Gytis Dudas^1,2,*^

^1^Institute of Biotechnology, Life Sciences Center, Vilnius University, Vilnius, Lithuania

^2^Hematology, Oncology and Transfusion Medicine Center, Vilnius University Hospital Santaros Klinikos, Vilnius, Lithuania

^3^Division of Virology, Department of Pathology, University of Cambridge, Addenbrooke’s Hospital Lab, CB2 2QQ, United Kingdom

^4^State Data Agency of Lithuania, Vilnius, Lithuania

^5^The Office of the Government of the Republic of Lithuania, Vilnius, Lithuania

^6^Department of Genomic Medicine, Rigshospitalet, Copenhagen, Denmark

^7^Erasmus University Medical Center, Rotterdam, the Netherlands

^8^Department of Anatomy and Physiology, Faculty of Veterinary Medicine, Lithuanian University of Health Sciences, Kaunas, Lithuania

^9^National Food and Veterinary Risk Assessment Institute, Vilnius, Lithuania

^10^State Food and Veterinary Service of Lithuania, Vilnius, Lithuania

^11^Veterinary Academy, Lithuanian University of Health Sciences, Kaunas, Lithuania

^12^Department of Experimental, Preventive and Clinical Medicine, State Research Institute Centre for Innovative Medicine, Vilnius, Lithuania

^13^Department of Genetics and Molecular Medicine, Hospital of Lithuanian University of Health Sciences Kauno Klinikos, Kaunas, Lithuania

^14^Institute of Cardiology, Lithuanian University of Health Sciences, Kaunas, Lithuania

^15^Department of Genetics and Molecular Medicine, Lithuanian University of Health Sciences, Kaunas, Lithuania

^16^Institute of Microbiology and Virology, Lithuanian University of Health Sciences, Kaunas, Lithuania

^17^Department of Laboratory Medicine, Lithuanian University of Health Sciences, Kaunas, Lithuania

^18^Department of Veterinary Pathobiology, Lithuanian University of Health Sciences, Kaunas, Lithuania

^*^Corresponding author. Institute of Biotechnology, Life Sciences Center, Vilnius University, Vilnius, Lithuania. E-mail: [gytis.dudas@gmc.vu.lt](mailto:gytis.dudas@gmc.vu.lt)

Abstract

Several studies have documented reverse zoonotic transmission of SARS-CoV-2, including in farmed mink which are susceptible to human respiratory viruses and are known for serving as a reservoir capable of generating new virus variants in densely populated farms. Here, we present the genomic investigation launched in response to detection of human infections with mink-origin SARS-CoV-2 lineages, and show evidence of at least 14 high-confidence introductions of SARS-CoV-2 from humans into farmed mink in Lithuania where sustained transmission in farmed mink lasted up to a year. We estimated the most likely timeframes for these introductions encompassing at least six SARS-CoV-2 lineages, some of which were already extinct in humans, with Bayesian phylogenetic and molecular clock analyses. This study highlights the public health risks posed by fur farms and underscores that passive genomic surveillance systems are ineffective without the active involvement and expertise of responsible institutions.

1. Introduction
   1. SARS-CoV-2 surveillance in mink farms in Lithuania

Early in the COVID-19 pandemic, infections were detected in mink farms in the Netherlands and later in other countries, confirming that mink are susceptible to SARS-CoV-2 (V’kovski et al. [2021](#B31); Sun et al. [2021](#B26)). Anthropozoonotic spillovers were also recorded in the Netherlands and Denmark, including transmission between farms (Jahid, Bowman, and Nolting [2024](#B8); Oreshkova et al. [2020](#B18)). In Lithuania, despite multiple officially recognised outbreaks on mink farms in late 2020, no mass culling was performed. It was only in November 2020, after outbreaks in Denmark, that the State Food and Veterinary Service (SFVS) initiated a passive surveillance program. This program was based on mandatory self-reporting by farmers of increased mink mortality or morbidity, as well as any confirmed SARS-CoV-2 cases among farm employees (Lithuanian parliament [2020](#B10), [2021a](#B11); Žigaitė et al. [2023](#B33)), but no periodic testing of farm animals to detect the infection.

The first two SARS-CoV-2-infected mink farms in Lithuania were identified in November and December 2020, with two more detected in early 2021 (Žigaitė et al. [2023](#B33)). Instead of mass culling, the SFVS developed an outbreak control plan. All SARS-CoV-2-positive or possibly infected mink were to be culled, including those in neighboring cages as the main measure to control the infection. Additionally, any movement of animals between farms required an SFVS permit once disease was detected (1991). From December 2021, prior to animal transfer, farms had to report their zoonotic disease status and any confirmed or suspected COVID-19 cases among employees. However, mandatory animal testing before transfers was not required, if the farm’s “zoonotic disease status” was considered acceptable by SFVS.

- 1. Human SARS-CoV-2 surveillance and lineage dynamics in Lithuania

Human SARS-CoV-2 genomic surveillance in Lithuania began with limited sampling. The first sequence from Lithuania was uploaded to GISAID in October 2020 (Khare et al. [2021](#B9)). Routine and representative surveillance in Lithuania was launched in March 2021. Available data indicate that from October 2020 to February 2021, i.e. prior to routine surveillance, B.1.177.60 and other B lineages (notably B.1.1.280) were the most prevalent in humans in Lithuania. Later, in March 2021, these lineages were pushed out by Alpha lineages (B.1.1.7, its sublineage Q.1 and their relatives). Alpha-like lineages stayed dominant until July 2021, when case numbers receded during the summer. Afterwards, Delta/B.1.617.2 drove a wave of COVID-19 cases that established these lineages as the predominant SARS-CoV-2 variant in the country (Delta-descendant AY.4.5 lineage in particular). Delta-like lineages stayed dominant until January 2022, when Omicron/B.1.1.529 variants swept the world, pushing other SARS-CoV-2 lineages previously circulating in humans to apparent extinction.

- 1. Evidence of mink-to-human SARS-CoV-2 spillover and passive surveillance

On 2021 October 05, at a time when the Delta/B.1.617.2 lineage was dominant globally (including in Lithuania), routine human SARS-CoV-2 surveillance discovered a mink farm worker infected with lineage B.1.343, which hadn’t been seen in humans in Lithuania since December 2020. A similar case of B.1.177.60, an extinct lineage of Lithuanian origin with mink-adaptive mutations from a farm worker, was detected a few weeks later (2021 November 11). As a result, a country-wide testing of mink farms was carried out in 2021 November–December. All 57 mink farms in the country were tested without prior warning and evidence of SARS-CoV-2 infection was detected in 25 farms via RT-PCR or ELISA (Žigaitė et al. [2023](#B33)).

- 1. Integrating mink and human genomic surveillance in Lithuania

Here, we present analyses of SARS-CoV-2 genomes recovered from mink in Lithuanian fur farms that tested positive during this country-wide sampling. We employ Bayesian phylogenetic analyses involving molecular clocks and ancestral state reconstruction to infer the number and timing of human-to-mink transmissions of SARS-CoV-2. By combining these data with national statistics, we connect these spillovers to population dynamics of farmed mink and SARS-CoV-2 in humans. In particular, we focus on two specific lineages - B.1.343 and B.1.177.60 - that sparked this investigation in the first place, because they represent the longest cryptically circulating SARS-CoV-2 lineages in Lithuania. This prolonged period of undetected circulation allows us to hypothesise about the mechanisms responsible for long-term persistence of SARS-CoV-2 in Lithuanian mink. Combined, our work highlights the weaknesses of passive veterinary surveillance, the advantages of genomic surveillance in One Health contexts (even when one host is undersampled), and the excessive public health risks posed by fur farming activities. Based on the circumstances surrounding our investigation, we speculate on a hypothetical mechanism for long-term SARS-CoV-2 persistence in mink farms in Lithuania.

We analyse all publicly available mink-derived SARS-CoV-2 genome sequences collected during the period when genomic surveillance data is available, spanning from the end of 2020 to the beginning of 2022. While mink farming continued beyond this period, routine genomic surveillance of SARS-CoV-2 in mink was never established in Lithuania and we have no sequenced SARS-CoV-2 genomes sampled from mink after this period, while human surveillance was scaled back to sampling from hospitals. As a result, our analyses are restricted to the period between late 2020 and early 2022, which nonetheless captures the period of documented mink infections and provides insight into the likely human-to-mink spillover events occurring during that time.

1. Methods
   1. SARS-CoV-2 samples and genomic surveillance in Lithuania

The SARS-CoV-2 genomic surveillance programme in Lithuania was launched in March 2021, combining sequencing capacities of Vilnius University (VU) Life Sciences Center, VU hospital Santaros Klinikos, Lithuanian University of Health Sciences (LUHS), LUHS hospital Kauno Klinikos, and the ECDC. The programme sequenced, on average, over 600 genomes per week, with targeted sequencing of all PCR-positive individuals working on mink farms (for further methodological details, see the supplementary section “SARS-CoV-2 genomic surveillance programme”). In contrast, the SFVS did not implement a dedicated genomic surveillance framework for mink, and sequencing of mink-origin samples was only conducted during confirmed farm outbreaks.

SARS-CoV-2 samples from mink, used in this article, were collected by SFVS and sequenced by the National Food and Veterinary Service of Lithuania, Erasmus University Medical Center performed sequencing for the most Lithuanian mink samples before active surveillance initiated in November 2021. Genome quality was re-evaluated and improved through reassembly with the COVID-19-SIGNAL pipeline (Nasir et al. [2024](#B16)), with 58 genomes assigned to 6 lineages (AY.4, AY.122, B.1.1.7, B.1.1.280, B.1.177.60, B.1.343). Details are provided in the supplementary section “Mink-origin SARS-CoV-2 genomes.”

Additional data, including COVID-19 case numbers, mink farm registry information, and herd sizes, were obtained from the National Data Agency of Lithuania and the Agriculture Data Center (State Agricultural Data Centre of Lithuania, n.d.; National data agency of Lithuania, n.d.).

- 1. Molecular clock analyses

Lineage-specific datasets were constructed for six SARS-CoV-2 lineages: B.1.1.7, AY.4, AY.122, B.1.177.60, B.1.1.280, and B.1.343. “Mink-adjacent” and broader contextual genomes were selected via UShER(Turakhia et al. [2021](#B30)) and GISAID(Shu and McCauley [2017](#B23)), incorporating both local and global sequences. Sampling was performed on a lineage-by-lineage basis, typically at 1–10 sequences per country per month depending on lineage prevalence, with targeted downsampling for overly large datasets. For low-prevalence lineages (B.1.1.280 and B.1.343), ancestral sequences (B.1.1 and B.1, respectively) were added. Dataset curation and circulation window constraints are described in the supplementary section “Contextual data” and Supplementary Table 2.

Phylogenetic inference was performed using BEAST v1.10.4, with the SRD06 (Shapiro, Rambaut, and Drummond [2006](#B22)) substitution model, uncorrelated relaxed molecular clock, and a Coalescent Bayesian SkyGrid tree prior. Host species (human or mink) was modeled as a discrete trait, with Bayesian stochastic search variable selection (BSSVS) and Markov jumps used to infer interspecies transmission events (Gill et al. [2013](#B6)). Five human-origin samples with mink-adaptive mutations were reclassified as mink to create a more accurate ancestral state reconstruction. Each analysis ran for 200 million MCMC steps, sampling every 20,000 steps. Most lineages used three replicate chains; B.1.1.280 required 11 runs due to bimodal posterior distributions, with six retained after filtering (see section “Post-processing of MCMC samples”).

All parameters were assessed in Tracer v1.7.2 (Rambaut et al. [2018](#B21)), with effective sample sizes (ESSs) > 200 considered acceptable. Final alignments were generated in Nextclade v3.8.2 against reference sequence NC_045512.2.

- 1. Calculation of human to mink transmission times

For each lineage, MCMC output trees were combined with LogCombiner (v1.10.4) with burn-in removed, corresponding to 10% of the generated output trees or 20 million initial trees, leaving 27 000 trees for analysis in each case (75% or 150 million initial trees for B.1.1.280, leaving 15 000 trees for analysis). From these combined trees, maximum credibility clade trees were generated with TreeAnnotator(v1.10.4). For each human-to-mink transmission event which was visually identified from the maximum clade credibility (MCC) tree, the most recent common ancestor (MRCA) was identified together with all the descendant leaves. For further analysis, MCMC output trees were used, by taking only the MRCA nodes with leaves identical to leaves in the MCC tree, i.e. conditioning our analysis on the clades present in the MCC tree that represent SARS-CoV-2 introductions into mink. From these clades, introduction times from humans into mink were extracted and summarised.

- 1. Visualisation and filtering

All visualisations were created using Python (3.12.8) and several packages (matplotlib 3.10.1, cartopy 0.24.0, shapely 2.1.0, baltic 0.3.0, pandas 2.2.3). SNP (single nucleotide polymorphism) alignments with phylogenetic trees were generated by extracting the subtree of interest from the MCC tree for each lineage.

- - 1. Case numbers and lineage proportions

Case numbers and lineage proportions were visualised with matplotlib (version 3.9.2) in Python. For a period from March to May and September to November in 2021, more than 6 million (6 376 146) antigen and PCR tests were performed in Lithuania, with a test positivity rate of 8.3% (527 082), and a rate of 3.3% (17 187) for sequencing. This time period covers the beginning of genomic surveillance in Lithuania in early 2021, capturing the tail-end of the winter wave and arrival of Alpha-like lineages (March to May), skips the summer with low COVID-19 case numbers and extremely high sequencing coverage, and resumes in late 2021 (September to November), encompassing the wave driven by Delta-like lineages and before the arrival of Omicron-like lineages. This avoids unusual periods of surveillance that may bias epidemic coverage estimates, as up to 25.6% of known COVID-19 cases were sequenced during the summer of 2021 and as few as 0.3% during the Omicron wave (averaging two-week periods). Given an adequate epidemic coverage, we assumed that sampled lineage proportions of SARS-CoV-2 genomes from Lithuania were representative of the whole population; as such, we scaled these lineage proportions to total SARS-CoV-2 case numbers in Lithuania. The lineage proportions and case numbers were aggregated into 14-day periods.

- - 1. Estimating the number and dates of human-to-mink transmissions

For each focal lineage analysis after discarding 20 million states as burn-in (with additional processing for lineage B.1.1.280, outlined above), we extracted the human-to-mink Markov jump (Minin and Suchard [2008](#B14)) dates from each tree sampled during MCMC. Since many of the human-to-mink transition dates were multimodal, we used an algorithm that uses a kernel density estimate (KDE) of the jump dates and simulates a vertically descending line that intersects the KDE at multiple points and computes the integral until it reaches the 95% highest posterior density interval. Following the same burn-in processing, we also extracted the number of human-to-mink Markov jump state changes from the posterior.

1. Results

We analysed 1 345 SARS-CoV-2 genomes from humans and 58 from mink to assess the effectiveness of Lithuania's genomic surveillance programme and to evaluate the risks associated with mink farming. We identified mink-associated human infections bearing mutations associated with adaptation to mink, commonly affecting mink farm employees and caused by SARS-CoV-2 lineages that have been extinct in the general human population for months.

- 1. Correcting ancestral state reconstruction

During initial runs, we noticed that three lineage B.1.343 spillback events into humans were strongly and incorrectly informing ancestral state reconstruction at neighbouring nodes (i.e. inferring internal nodes of the mink-associated subtree as human). There is abundant evidence that this is incorrect: 1) the last time lineage B.1.343 was seen in the human population of Lithuania was in December 2020 (EPI_ISL_934083, recovered in Vilnius city municipality as part of opportunistic sampling at a hospital), while its reemergence in May 2021 (EPI_ISL_2428882) and October 2021 (EPI_ISL_5390697) happens at a time when major global variants of concern (VOCs) Alpha and Delta had outcompeted virtually all co-circulating SARS-CoV-2 variants; 2) two of the samples (EPI_ISL_5390697, EPI_ISL_2428882) contain the mink-associated ORF3a:L219V (Elaswad et al. [2020](#B5); Naderi et al. [2023](#B15)) mutation, and all three samples contain S:F486L and ORF1a:G4177E mutations associated with mink adaptation (Elaswad et al. [2020](#B5); Naderi et al. [2023](#B15); Su et al. [2022](#B25)), and 3) none of these mutations are detected in an earlier human sample from December 2020 (EPI_ISL_934083)**,** suggesting that this lineage got into mink populations at a later point.; and 3) all post-2020 B.1.343 cases in Lithuania were employed in mink farms.

A similar situation was noticed for lineage B.1.177.60, where the last human infections with this lineage in Lithuania were detected in June 2021 (EPI_ISL_3060127) and subsequent detections were in mink farmers or mink in November 2021, at a time when Delta variants were dominant. Additionally, the mink-associated ORF3a:L219V mutation is present in both human and mink samples from November 2021. At the time when these suspected zoonotic spillover events were detected in the Lithuanian population, there were no reported sequences of lineages B.1.343 and B.1.177.60 anywhere in the world (Figure 1).

In both cases, continued circulation of SARS-CoV-2 in mink rather than humans is more parsimonious. For lineage B.1.343, the Lithuanian SARS-CoV-2 genomic surveillance programme sequenced 21 661 SARS-CoV-2 genomes out of 508 942 human cases between 2020 December 11 (last known human B.1.343 infection, EPI_ISL_934083) and 2021 October 05 (first putative spillback from mink into humans, EPI_ISL_5390697) which corresponds to a maximum probable prevalence of 1.17×10^-4^ in the absence of lineage detection (Brito et al. [2022](#B2)). Maximum probable prevalence was estimated under a binomial proportion confidence interval using Jeffreys method (Beta(1/2, 1/2) prior), with the upper 95% credible bound calculated for zero observed detections (Brito et al. [2022](#B2)), i.e. it estimates how common, at most, a lineage can be in the absence of detections (with 95% confidence) under a given sample size. For SARS-CoV-2 lineage B.1.343 to have sustained itself over this nearly year-long period exclusively via human-to-human transmission and without detection, it could have caused, at most, 59 human cases over 298 days. For lineage B.1.177.60, the equivalent calculation is: last detected human case 2021 June 23 (EPI_ISL_3060127), first putative spillback 2021 November 11 (EPI_ISL_7082794) with 10 403 SARS-CoV-2 genomes sequenced from 341 808 human cases over this time without detections and therefore a maximum probable prevalence of 2.41×10^-4^ in the human population. For sustained and undetected human-to-human transmission this would require around 83 human cases over 141 days which is also questionable. The multiple associations of these lineages with mink (detection in mink, spillover into mink farm workers, mink-adaptive mutations), and in the absence of “saltation” seen in many chronic immunosuppressed human infections (Supplementary Figures S10 and S11), the host trait for sequences (S21L465|Lithuania (EPI_ISL_7083492), S21L477|Lithuania (EPI_ISL_7082794), S21E887|Lithuania (EPI_ISL_2428956), S21E881|Lithuania (EPI_ISL_2428882), IBT-LCS-VU_r24_28|Lithuania (EPI_ISL_5390697) was assigned as “mink” rather than “human” for BEAST analysis.

- 1. Mink population dynamics in Lithuanian farms

Neither the farmed mink nor the spread of SARS-CoV-2 during the COVID-19 pandemic were markedly impacted by public health responses (Figure 2). Mink farming is seasonal with known breeding and pelting times, leading to regular population spikes. The decrease in mink population before 2020 may be associated with decreased pelt prices and subsequent increase in population prior to 2021 could be associated with increased pelt prices, potentially affected by mink culling in Denmark and the Netherlands, i.e. decreased global pelt supply. Gradual annual decrease of the mink population since 2021 can be attributed to the ban of fur farms (first proposed in the parliament in November 2021 (Lithuanian parliament [2021b](#B12))) in Lithuania that is supposed to take effect in 2027 (Lithuanian parliament [2023](#B13)).

- 1. Lineages transmitted from humans to mink
     1. B.1.1.280 and B.1.343

Routine SARS-CoV-2 surveillance started only after B.1.1.280 and B.1.343 went extinct in humans (Figure 3). B.1.1.280 was a largely Lithuania-restricted pre-Alpha lineage circulating in mid-to-late 2020 while B.1.343 was an obscure lineage mostly circulating in Denmark in 2020. These lineages were initially detected in Lithuania during sporadic sequencing efforts in September 2020 and December 2020 (Dudas et al. [2021](#B4)). Mink-associated B.1.343 cases appeared in late 2021, both in mink and mink farmers but not in the general human population. Notably, there are two infected mink farm worker cases of B.1.343 in May 2021 that were not identified as mink-associated by the SARS-CoV-2 genomic surveillance programme in Lithuania at the time due to co-circulation of non-VOC lineages and only identified as such in October 2021, after the third spillback was discovered. By analysing posterior outputs from BEAST we infer that human-to-mink spillover for lineage B.1.343 happened once between October and December 2020 (Figure 3, Figure 4, Supplementary Figure S8, Supplementary Table 1). The mink farm worker cases discovered to be infected with lineage B.1.343 also contained some known adaptations to mink (ORF3a:L219V, S:F486L, ORF1a:G4177E) and were phylogenetically close to genomes from mink (Figure 5). Similarly, lineage B.1.1.280 also seems to have spilled over once, between June and September 2020 (Figure 3, Figure 4, Supplementary Table 1) when the farmed mink population was high.

- - 1. B.1.177.60

Lineage B.1.177.60 circulated in the Lithuanian population from October 2020 to March 2021, and was a locally prevalent descendant of a common European lineage B.1.177 (Hodcroft et al. [2021](#B7)). We estimate at least two spillover events from humans to mink between November 2020 and February 2021 (Figure 3, Figure 4, Supplementary Table 1) and between December 2020 and November 2021 (Figure 3, Figure 4, Supplementary Figure S9**,** Supplementary Table 1). Although no human-to-human transmission of this lineage was observed in the general Lithuanian human population in late 2021, B.1.177.60 infections were identified in both mink and mink farm workers. Some sequences in this clade from human infections contain known mink-adaptive mutations (e.g., ORF3a: L219V, S: Y453F (Tan et al. [2022](#B29))).

- - 1. B.1.1.7

Lineage B.1.1.7 was prevalent in the Lithuanian population from January to June 2021, and we estimate at least two introductions of it from humans to mink between January 2021 and August 2021 (Figure 3, Figure 4, Supplementary Figure S6, Supplementary Table 1) and between November 2020 and December 2021 (Figure 3, Figure 4, Supplementary Figure S7, Supplementary Table 1)

- - 1. AY.4 and AY.122

Both lineages AY.4 and AY.122 circulated in the Lithuanian population from July 2021 to Jan 2022. We estimate at least seven (Figure 3, Figure 4, Supplementary Table 1) anthroponotic transmissions from humans to farmed mink of lineage AY.4 and one for AY.122 (Figure 3, Figure 4, Supplementary Table 1). For AY.4, the 95% HPD regions, while overlapping, span from July 2021 to May 2022 with almost all of these jumps clustered around October 2021, at the time when AY.4 and descendant lineages were most common in humans. As for AY.122 lineage, the 95% highest posterior density region for the human to mink jump event is around September to October 2021, also coinciding with high numbers of SARS-CoV-2 infections in humans and peak farmed mink population within the year (Figure 3). We suspect that the volume of introductions of Delta-related lineages into mink is not inherently unique or reflects any changes in the underlying epidemiology of SARS-CoV-2 in either humans or mink but captures a period of time when Delta-like lineages were common in humans in Lithuania and mink were under intensive surveillance.

- 1. Mink-to-human transmissions

We identified likely four independent SARS-CoV-2 mink-to-human transitions by inspecting MCC trees (Figure 5, Supplementary folder “MCC_Trees”): At least one for B.1.177.60 and most likely three for B.1.343 lineage. For B.1.177.60, two human samples (S21L477|Lithuania and S21L465|Lithuania) were sampled on the same day (2021-Nov-11) and it is impossible to determine if one of the infections was human-to-human, but they differ by one nucleotide (and one additional compatible but ambiguous nucleotide position) and are phylogenetically closely related to the 12126|Lithuania (EPI_ISL_10571267) sequence obtained from farmed mink (2021-Nov-04) (Figure 5). Human cases of B.1.343 in 2021, however, are not closely related, indicating the occurrence of multiple mink-to-human transmissions. Overall, the real number of mink-to-human jumps remains unclear as we have very limited sequence data from mink, especially close to their suspected transmission times. We have very limited sampling of mink farm workers: only 29 sequences are derived from mink farm workers, of which five (17.24%) are associated with mink infections. Based on these limited data, mink farm workers had 1 : 4.80 odds in favour of being infected by another human (0.8276 of infections) than mink. Accounting for the small sample size using a binomial proportion confidence interval (Clopper-Pearson method) the 95% confidence interval for proportion of infections being mink-associated ranges between 0.058 and 0.358, corresponding to odds of roughly 1 : 1.80 to 1 : 16.11 in favour of human-origin infections in mink farm workers. As can be seen in the AY.4 lineage group example (Figure 3; Figure 4; Supplementary Figures S1-S4) active veterinary surveillance readily detects multiple human-origin SARS-CoV-2 lineages in mink when these lineages are common in humans.. Therefore, we expect a considerable number of mink infections with SARS-CoV-2 to have gone undetected, due to limited genomic surveillance efforts.

- 1. Omicron lineages and further surveillance

Soon after the one pulse of active country-wide veterinary surveillance on mink farms for SARS-CoV-2, Omicron (B.1.1.529) and its descendants swept through the human population. In the absence of additional SARS-CoV-2 surveillance in mink after 2021, we do not have data to determine if Omicron-related lineages also entered mink populations and/or were able to establish and adapt to the mink populations.

1. Discussion

Human-to-mink transmission of SARS-CoV-2, sustained circulation in mink, and occasional spillback into humans have been repeatedly documented during the COVID-19 pandemic (Jahid, Bowman, and Nolting 2024; Oreshkova et al. [2020](#B18); Domańska-Blicharz et al. [2023](#B3); Rabalski et al. [2022](#B20)). Using whole-genome sequencing from Lithuania’s routine human genomic surveillance alongside a one-off pulse of active veterinary surveillance in mink, we inferred the timing, frequency, and persistence of cross-species transmission events of SARS-CoV-2 detected between 2021 and 2022 (Figures 3 and 4). Closely related viruses were detected in mink farm workers and carried mink-adaptive mutations (e.g., S:Y453F, S:F486L, S:N501T, ORF3a:L219V) (Figure 5), supporting mink-to-human transmission (28,31). These mutations are not associated with increased fitness in humans and may incur fitness tradeoffs favoring mink (Naderi et al. [2023](#B15); Zhou et al. [2022](#B32)). Root-to-tip regressions do not show common signatures of chronic human infections (Figures S10 and S11) and the extinction of these lineages from the human population, combined with extensive associations with mink (related lineages seen in mink previously, circulation on multiple mink farms, occupation of infected persons, and mutation profile of SARS-CoV-2) makes alternative reservoirs or undetected human circulation unlikely, suggesting animal movement as the main mechanism of B.1.343 and B.1.177.60 transmission between herds.

Notably, the most numerous introductions of SARS-CoV-2 from humans into mink happened during active veterinary surveillance in late 2021, predominantly involving Delta lineages prevalent in the Lithuanian human population at the time. We speculate that spillover from humans into mink is likely to be frequent in Lithuania, but only rarely results in prolonged circulation, with B.1.343 and B.1.177.60 lineages being those rare examples. These two lineages circulated undetected in mink for nearly a year and were only identified after spillback into humans through active human surveillance.

Overall, mink farming represents a substantial risk for zoonotic emergence and viral evolution, effectively constituting unsanctioned gain-of-function experiments that would be unacceptable in laboratory settings (Peacock and Barclay [2023](#B19)). In Lithuania, farmers had no incentives to report outbreaks in mink farms (Arūnas Milašius [2020](#B1)), decisive interventions (e.g. culling of affected farms) were not tied to specific epidemic thresholds and thus never undertaken, active veterinary surveillance in mink farms was never routine, passive surveillance for detecting sustained circulation in farms was insufficient (Žigaitė et al. [2023](#B33); Domańska-Blicharz et al. [2023](#B3)) and little was done to communicate about the dangers of continued SARS-CoV-2 circulation in animals to the public. Our study demonstrates that repeated human-to-mink spillovers, prolonged undetected circulation in mink, and subsequent spillback into humans occurred in Lithuania, exposing critical weaknesses in passive surveillance and intersectoral coordination. Addressing these failures is essential for mitigating zoonotic risks and improving preparedness for future pandemics.

Acknowledgments

This project has received funding from the Research Council of Lithuania, Lithuania under the EMBO Installation Grant programme under grant No 5305 (GD).

Data availability

Main and supplementary figures, BEAST XML (with sequences removed, per GISAID’s user agreement), trees (posterior and MCC), and log files, data on mink and human SARS-CoV-2 case data, GISAID acknowledgment tables and accessions used, as well as code to analyse and visualise data are available on Zenodo: <https://zenodo.org/records/16417485>.

References

Milašius A. Koronavirusą Aptikusio Audinių Ūkio Savininkas: Nuo Valstybės Priklausys, Ar Nebus Imta Slėpti Tokių Atvejų. *News portal, DelfiLt (Delfi, 26 November)* 2020. <https://www.delfi.lt/agro/agroverslo-naujienos/koronavirusa-aptikusio-audiniu-ukio-savininkas-nuo-valstybes-priklausys-ar-nebus-imta-slepti-tokiu-atveju-85840055?fbclid=IwZXh0bgNhZW0CMTAAAR1dZEqWLcHyI2wu28E9xe2fPudSyxvVExiqDx1hK-qeQn0XVa23lcR18Ag_aem_nTz71SepHWjCxL2kPBoKHA>, accessed 16 May 2025

Brito AF, Semenova E, Dudas G et al. Global disparities in SARS-CoV-2 genomic surveillance. *Nat Commun* 2022;13:7003. [10.1038/s41467-022-33713-y](https://doi.org/10.1038/s41467-022-33713-y)

Domańska-Blicharz K, Oude Munnink BB, Orłowska A et al. Cryptic SARS-CoV-2 lineage identified on two mink farms as a possible result of long-term undetected circulation in an unknown animal reservoir, Poland, November 2022 to January 2023. *Eurosurveillance* 2023;28:2300188. [10.2807/1560-7917.ES.2023.28.16.2300188](https://doi.org/10.2807/1560-7917.ES.2023.28.16.2300188)

Dudas G et al. Emergence and spread of SARS-CoV-2 lineage B. 1.620 with variant of concern-like mutations and deletions. *Nat Commun* 2021;12/1:5769.

Elaswad A, Fawzy M, Basiouni S et al. Mutational spectra of SARS-CoV-2 isolated from animals. *PeerJ* 2020;8:e10609. [10.7717/peerj.10609](https://doi.org/10.7717/peerj.10609)

Gill MS, Lemey P, Faria NR et al. Improving Bayesian population dynamics inference: A coalescent-based model for multiple loci. *Mol Biol Evol* 2013;30:713–24. [10.1093/molbev/mss265](https://doi.org/10.1093/molbev/mss265)

Hodcroft EB, Zuber M, Nadeau S et al. Spread of a SARS-CoV-2 variant through Europe in the summer of 2020. *Nature* 2021;595:707–12. [10.1038/s41586-021-03677-y](https://doi.org/10.1038/s41586-021-03677-y)

Jahid MJ, Bowman AS, Nolting JM. SARS-CoV-2 outbreaks on mink farms—A review of current knowledge on virus infection, spread, Spillover, and containment. *Viruses* 2024;16:81. [10.3390/v16010081](https://doi.org/10.3390/v16010081)

Khare S, Gurry C, Freitas L et al. GISAID’s role in pandemic response. *China CDC Weekly* 2021;3:1049–51. [10.46234/ccdcw2021.255](https://doi.org/10.46234/ccdcw2021.255)

Lithuanian parliament. Lithuanian law B1-850. *Law database, E-Seimas (Lithuanian parliament, 7 November)* 2020. <https://e-seimas.lrs.lt/portal/legalAct/lt/TAD/5f1762d0207611eb9604df942ee8e443?positionInSearchResults=3&searchModelUUID=8c2c6270-d222-47d3-96e3-9faf685dd367>, accessed 16 May 2025

Lithuanian parliament. Lithuanian law B1-991. *Law database, E-Seimas (Lithuanian parliament, 18 December)* 2021a. <https://e-seimas.lrs.lt/portal/legalAct/lt/TAD/1badcfa25f7911ecb2fe9975f8a9e52e?positionInSearchResults=0&searchModelUUID=0cc667be-b77d-4de5-9bf2-1ee5f5467191>, accessed 16 May 2025

Lithuanian parliament. Media report on mink farm ban proposal in the parlament. *Lithuanian parliament website, lsrlt (Lithuanian parliament, 15 November)* 2021b. <https://www.lrs.lt/sip/portal.show?p_r=40564&p_k=1&p_t=278912>, accessed 10 July 2025

Lithuanian parliament. Lithuanian law VIII-500. *Law database, E-Seimas (Lithuanian parliament)* 2023. <https://e-seimas.lrs.lt/portal/legalAct/lt/TAP/1cd60150010711eebc0bd16e3a4d3b97>, accessed 2 July 2025

Minin VN, Suchard MA. Counting Labeled transitions in continuous-time Markov models of evolution. *J Math Biol* 2008;56:391–412. [10.1007/s00285-007-0120-8](https://doi.org/10.1007/s00285-007-0120-8)

Naderi S, Chen PE, Murall CL et al. Zooanthroponotic transmission of SARS-CoV-2 and host-specific viral mutations revealed by genome-wide phylogenetic analysis. *Elife* 2023;12:e83685. [10.7554/eLife.83685](https://doi.org/10.7554/eLife.83685)

Nasir JA, Maguire F, Smith KM et al. SARS-CoV-2 Illumina GeNome assembly line (SIGNAL), a Snakemate workflow for rapid and bulk analysis of Illumina sequencing of SARS-CoV-2 genomes. *NAR Genomics and Bioinformatics* 2024;6:lqae176. [10.1093/nargab/lqae176](https://doi.org/10.1093/nargab/lqae176)

National data agency of Lithuania. COVID-19 Lithuanian open statistics portal. *Open statistics portal, COVID-19 Lietuvoje* n.d.. <https://osp.stat.gov.lt/covid-dashboards>, accessed 17 May 2025

Oreshkova N, Molenaar RJ, Vreman S et al. SARS-CoV-2 infection in farmed minks, the Netherlands, April and may 2020. *Eurosurveillance* 2020;25:2001005. [10.2807/1560-7917.ES.2020.25.23.2001005](https://doi.org/10.2807/1560-7917.ES.2020.25.23.2001005)

Peacock TP, Barclay WS. Mink farming poses risks for future viral pandemics. *Proc Natl Acad Sci* 2023;120:e2303408120. [10.1073/pnas.2303408120](https://doi.org/10.1073/pnas.2303408120)

Rabalski L, Kosinski M, Mazur-Panasiuk N et al. Zoonotic spill-over of SARS-CoV-2: Mink-adapted virus in humans. *Clin Microbiol Infect* 2022;28:451.e1–4. [10.1016/j.cmi.2021.12.001](https://doi.org/10.1016/j.cmi.2021.12.001)

Rambaut A, Drummond AJ, Xie D et al. Posterior summarization in Bayesian phylogenetics using tracer 1.7. *Syst Biol* 2018;67:901–4. [10.1093/sysbio/syy032](https://doi.org/10.1093/sysbio/syy032)

Shapiro B, Rambaut A, Drummond AJ. Choosing appropriate substitution models for the phylogenetic analysis of protein-coding sequences. *Mol Biol Evol* 2006;23:7–9. [10.1093/molbev/msj021](https://doi.org/10.1093/molbev/msj021)

Shu Y, McCauley J. GISAID: Global initiative on sharing all influenza data–from vision to reality. *Eurosurveillance* 2017;22:30494. [10.2807/1560-7917.ES.2017.22.13.30494](https://doi.org/10.2807/1560-7917.ES.2017.22.13.30494)

State Agricultural Data Centre of Lithuania. Mothly reports of agricultural statistics. *Data portal, State Agricultural Data Centre of Lithuania* n.d.. <https://zudc.lt/ukiniu_gyvunu_registras/menesio-ataskaitos/>, accessed 17 May 2025

Su C, He J, Han P et al. Molecular basis of mink ACE2 binding to SARS-CoV-2 and its mink-derived variants. *J Virol* 2022;96:e0081422–2. [10.1128/jvi.00814-22](https://doi.org/10.1128/jvi.00814-22)

Sun H, Li F, Liu Q et al. Mink is a highly susceptible host species to circulating human and avian influenza viruses. *Emerging Microbes & Infections* 2021;10:472–80. [10.1080/22221751.2021.1899058](https://doi.org/10.1080/22221751.2021.1899058)

Suomen Turkiseläinten Kasvattajain Liitto (STKL) ry. Fifur statistics. 2025. <https://fifur.fi/turkiselinkeinon-tilastot>, accessed 8 July 2025

Supreme Council of the Republic of Lithuania – Reconstituent Seimas. Veterinary law I-2110. *Law database, e-seimas (17 December)* 1991. <https://www.e-tar.lt/portal/lt/legalAct/TAR.97BDCD719E57/asr>, accessed 8 July 2025

Tan CCS, Lam SD, Richard D et al. Transmission of SARS-CoV-2 from humans to animals and potential host adaptation. *Nat Commun* 2022;13:2988. [10.1038/s41467-022-30698-6](https://doi.org/10.1038/s41467-022-30698-6)

Turakhia Y, Thornlow B, Hinrichs AS et al. Ultrafast sample placement on existing tRees (UShER) enables real-time phylogenetics for the SARS-CoV-2 pandemic. *Nat Genet* 2021;53:809–16. [10.1038/s41588-021-00862-7](https://doi.org/10.1038/s41588-021-00862-7)

V’kovski P, Kratzel A, Steiner S et al. Coronavirus biology and replication: Implications for SARS-CoV-2. *Nat Rev Microbiol* 2021;19:155–70. [10.1038/s41579-020-00468-6](https://doi.org/10.1038/s41579-020-00468-6)

Zhou J, Peacock TP, Brown JC et al. Mutations that adapt SARS-CoV-2 to mink or ferret do Not increase fitness in the human airway. *Cell Rep* 2022;38:110344. [10.1016/j.celrep.2022.110344](https://doi.org/10.1016/j.celrep.2022.110344)

Žigaitė S, Masiulis M, Bušauskas P et al. Evaluation of SARS-CoV-2 passive surveillance in Lithuanian mink farms, 2020–2021. *Frontiers in Veterinary Science* 2023;10:1181826. [10.3389/fvets.2023.1181826](https://doi.org/10.3389/fvets.2023.1181826)

**Figure** **1 Weekly counts of B.1.343 and B.1.177.60 SARS-CoV-2 genomes globally and in Lithuania.** The number of B.1.343 and B.1.177.60 lineage genomes globally (top) and in Lithuania (bottom), based on data from GISAID, filtered for only human genomes. Bars correspond to weekly counts.

**Figure** **2 Mink population dynamics over time in Lithuania.** The total mink population in Lithuanian mink farms with average yearly pelt price (auctions usually happen in march, June/July and September) on the regionally largest Saga furs market (top) and the average mink herd size, as well as breeding with slaughter seasons in Lithuania from 2017 to 2024 (bottom). The number of animals is indicated on the Y-axis, and time is indicated on the X-axis. Data is registered monthly. Price data from FiFur (Suom. Turk. Kasvattajain Liitto STKL Ry [2025](#B27)).

**Figure** **3. SARS-CoV-2 case trends, introduction events into mink and mink population dynamics in Lithuania. a)** all reported SARS-CoV-2 cases in humans in Lithuania, scaled by lineage frequencies. Lineages are grouped into lineage groups (described in methods), the hatched area represents the time span prior to the establishment of routine SARS-CoV-2 genomic surveillance system in the country. **b)** the 95% highest posterior density (HPD) intervals for estimated dates of SARS-CoV-2 introductions into mink, conditioned on events detected in maximum credibility clade trees. The coloured line continuing after the 95% HPD signifies the continued circulation period until the last detected sample, which is marked by the dot at the end of the line. The text below each line marks the mean circulation period in days with 95% HPD range, number of leaves and the posterior probability for a given node (does not apply if there was only a single sample in mink for a transmission event). **c)** the average mink population per herd in Lithuania over the same time period.

**Figure** **4 Posterior estimates of SARS-CoV-2 human-to-mink cross-species transmissions in Lithuania**. The numbers of transitions (Y-axis) are based on Markov jumps from all generated MCMC output trees after burn-in removal, and coloured by lineage. The width of the coloured bar represents posterior probabilities of each number of introductions. The bars outlined in black encompass the 95% highest probability mass. AY.4 lineages exhibit the highest number of human-to-mink transmissions, with all other lineages having 1-3 detected anthropozoonotic jumps.

**Figure** **5 SNP alignments with phylogenetic trees.** Starting from the left: A condensed alignment (described in methods) of polymorphic sites is displayed, keeping only the mutations causing amino acid changes or deletions in mink-derived SARS-CoV-2 genomes for lineages B.1.177.60 (**a**) and B.1.343 (**b**). Further to the right, subtrees containing the mink samples, extracted from MCC trees (with the closest contextual samples), posteriors indicated at nodes if value is higher than 0.5. Genomes in the alignment and tree are ordered the same. A map of Lithuania is shown on the right with lines connecting each tip to a random point within the municipality where the sample was collected. Tips are coloured according to sample type: Mink farm worker (yellow), general Lithuanian population (green), mink (red), or sequences outside of Lithuania (gray), see legend (upper right corner). The coats of arms of municipalities where samples were collected are displayed below the tree with municipalities coloured same as the background of each municipal coat of arms.

Supplementary Figure S1

Supplementary Figure S2

Supplementary Figure S3

Supplementary Figure S4

Supplementary Figure S5

Supplementary Figure S6

Supplementary Figure S7

Supplementary Figure S8

Supplementary Figure S9

Supplementary Figure S10

Supplementary Figure S11

Supplementary Table 1

Supplementary Table 2
